# *Arctium lappa* and *Arctium tomentosum*, Sources of *Arctii radix*: Comparison of Anti-Lipoxygenase and Antioxidant Activity as well as the Chemical Composition of Extracts from Aerial Parts and from Roots

**DOI:** 10.3390/plants10010078

**Published:** 2021-01-02

**Authors:** Weronika Skowrońska, Sebastian Granica, Magdalena Dziedzic, Justyna Kurkowiak, Maria Ziaja, Agnieszka Bazylko

**Affiliations:** 1 Department of Pharmacognosy and Molecular Basis of Phytotherapy, Faculty of Pharmacy, Medical University of Warsaw, Banacha 1, 02-097 Warsaw, Poland; weronika.skowronska@wum.edu.pl (W.S.); sebastian.granica@wum.edu.pl (S.G.); 2 Student’s Scientific Association at the Department of Pharmacognosy and Molecular Basis of Phytotherapy, Faculty of Pharmacy, Medical University of Warsaw, Banacha 1, 02-097 Warsaw, Poland; magda.dziedzic.m@gmail.com; 3 Department of Physical Chemistry, Faculty of Pharmacy, Medical University of Warsaw, Banacha 1, 02-097 Warsaw, Poland; justyna.kurkowiak@wum.edu.pl; 4 Institute of Physical Culture Studies, Rzeszów University, Cicha 2a, 35-326 Rzeszów, Poland; mziaja@ur.edu.pl

**Keywords:** *Arctium lappa*, *Arctium tomentosum*, caffeic acid derivatives, lipoxygenase, antioxidant, HPLC–DAD–MS^n^, total phenolic content

## Abstract

*Arctium lappa* is a weed used in traditional medicine in the treatment of skin inflammation and digestive tract diseases. *Arctium tomentosum* is used in folk medicine interchangeably with *Arctium lappa* and, according to European Medicines Agency (EMA) monography, provides an equal source of *Arctii radix* (*Bardanae radix*), despite the small amount of research confirming its activity and chemical composition. The aim of the study was the comparison of the anti-lipoxygenase and the antioxidant activity, scavenging of 2,2-diphenyl-1-picrylhydrazyl (DPPH), superoxide anion (O_2_^•−^), and hydrogen peroxide (H_2_O_2_), of 70 % (*v/v*) ethanolic extracts from the aerial parts and the roots of *Arctium lappa* and *Arctium tomentosum*. In the tested extracts, the total polyphenols content and the chemical composition, analyzed with the HPLC–DAD–MS^n^ method, were also compared. The extracts were characterized by strong antioxidant properties, but their ability to inhibit lipoxygenase activity was rather weak. A correlation between the content of polyphenolic compounds and antioxidant activity was observed. The extracts from *A. lappa* plant materials scavenged reactive oxygen species more strongly than the extracts from *A. tomentosum* plant materials. Moreover, the extracts from *A. lappa* plant materials were characterized by the statistically significantly higher content of polyphenolic compounds.

## 1. Introduction

*Arctium lappa* L., commonly known as the greater burdock, is a species from the Asteraceae family that grows in Europe, Asia, and North America [1]. It is a biennial plant, flowering from July to October. It grows commonly in Poland, especially in the ruderal places, near the water reservoirs, roadsides, and outbuildings [2].

Burdock plant is very popular in East Asian cuisine. It is harvested and eaten as a root vegetable, but its immature flowers, stalks, and young leaves are also used as food. In the United Kingdom burdock, is an ingredient in a popular soft drink, Dandelion and Burdock [3,4]. *Arctium lappa* is a beneficial component of the diet, mainly due to the content of many polyphenolic compounds, known for their health-promoting properties [5,6,7]. In folk medicine, mainly burdock roots, but also leaves and fruits, are used. The extracts from roots and leaves of burdock improve metabolism and diuretic action. They are used in catarrh of the gastrointestinal tract and to improve the liver and pancreas functions. In addition, they are recommended in the early stage of diabetes as a supportive means to maintain the proper level of glucose in the blood. Burdock fruits are used in traditional medicine as an aid in the treatment of cancer. Burdock leaves and roots are also used externally to relieve inflammation of the skin, such as pimples, boils, acne, and ulcers. They alleviate irritations and promote healing of wounds, including infected ones. When applied to the scalp, they prevent hair loss and act against dandruff [2,8,9].

In recent years, many studies have been carried out on the activity and chemical composition of extracts from roots, leaves, and fruits of greater burdock. In the extracts of burdock fruits, the presence of compounds from the group of lignans was found: lappaol A, lappaol C, lappaol F, matairesinol, arctiin, arctigenin, and arctigenic acid. In extracts of burdock leaves arctiin and arctigenin from the group of lignans, onopordopicrin from the group of sesquiterpene lactones and also flavonoids—luteolin, quercetin, quercitrin and rutin, and phenolic acids—were found. Burdock roots extracts are rich in phenolic acids—primarily dicaffeoylquinic acid isomers and their derivatives. They are also a rich source of polysaccharides, mainly inulin, phenolics, and polyacetylenes, and derivatives of unsaturated fatty acids—linoleate and oleate [10,11,12,13,14,15,16,17,18,19,20,21].

The antioxidant and the anti-inflammatory activity of *A. lappa* leaves, fruits, and roots, as well as individual compounds isolated from them, has been confirmed in studies in cell-free systems, on specific cell lines and using animal models [22,23,24,25]. It has been shown that the extract from greater burdock roots significantly reduced the time of regeneration of damaged skin [26,27]. *A. lappa* leaf extract affects the regulatory activity of melanogenesis by inhibiting melanin secretion [28]. The extract from burdock fruits stimulated collagen neogenesis in human fibroblasts [29]. Extracts from *A. lappa* roots administrated orally showed gastroprotective [30] and hepatoprotective effects [31,32]. Their beneficial effects in the treatment of peptic ulcer disease have been proven [33,34]. Burdock essence alleviates damage of the gastrointestinal tract mucosa and limits the growth of *H. pylori* [15,35,36]. In addition, it helps to maintain normal blood glucose level and have a beneficial effect on the lipid profile [25,37,38,39]. Polysaccharides isolated from the roots of *Arctium lappa* have been shown to have beneficial effects on the inhibition of proinflammatory cytokines in colitis in mice [40,41]. The antimicrobial activity of phenol-rich fractions from the extracts of burdock leaves has also been confirmed [42,43]. Some of the studies indicate that burdock extracts can be a promising neuroprotective agent. They act as protective in neurodegenerative diseases associated with oxidative stress [44,45,46]. 

*Arctium tomentosum* Mill., commonly known as a woolly burdock, occurs in Poland, often in ruderal places, and looks similar to *Arctium lappa*, but the flower head is covered densely in cobwebby hairs [47]. In traditional medicine it is used interchangeably with greater burdock. It is used to treat skin inflammations, relieves stomach discomfort, and has a diuretic effect [2,9]. *Arctium tomentosum* is mentioned in the monograph of the European Medicines Agency as a species providing an equivalent plant material with *Arctium lappa*–*Arctii radix (Bardanae radix)* [48]. Although in available sources the activity of woolly burdock extracts is comparable to the activity of greater burdock extracts [49,50], there are no comparative studies on the activity of both species. The chemical composition of *A. tomentosum* extracts has not been widely analyzed so far. It has been confirmed that arctiin and arctigenin were found in extracts from its fruits [51]. From methanolic extracts of *A. tomentosum* leaves and inflorescences, few flavonoids (kaempferol, quercetin, luteolin, and apigenin derivatives), chlorogenic acid, as well as lupeol 3-acetate and β-sitosterol 3-*O*-glucoside, were isolated. Moreover, in lipophilic extracts, the content of phytosterols, tocopherols, and β-amyrin was determined, and the composition of fatty acids was examined [52].

The aim of the study was to examine, in cell-free systems, anti-inflammatory activity (analyzed by the effect on lipoxygenase activity) and antioxidant properties (determined by the scavenging activity against synthetic radical, DPPH; superoxide anion, O_2_^•−^ and hydrogen peroxide, H_2_O_2_) of *Arctium lappa* and *Arctium tomentosum* aerial parts and roots extracts. The activity of extracts of one species obtained from the aerial parts and the roots from various natural positions of the Subcarpathian province was compared. The activity of extracts from aerial parts and the activity of roots extracts was also analyzed. A comparative analysis of the activity of extracts from the aerial parts and from the roots of *Arctium lappa* and *Arctium tomentosum* species was carried out. The total content of phenolic compounds in the tested extracts was determined using the Folin–Ciocalteu reagent. Then a comparative analysis between the activity and the content of phenolic compounds was carried out. Finally, the chemical profiles of investigated extracts were established with high-performance liquid chromatography coupled with mass spectrometry.

According to the monography of the European Medicines Agency, *Arctii radix (Bardanae radix)* is traditionally used as a diuretic in diseases of the urinary tract, as a stimulant of gastric secretion in temporary loss of appetite and as a treatment for seborrheic skin conditions [48]. It can be obtained from *Arctium lappa*, *Arctium minus*, *Arctium tomentosum,* and related species, hybrids, or mixtures thereof. Considering this, comparison of the chemical composition, as well as the activity of extracts from the aerial parts and from the roots of *Arctium lappa* and *Arctium tomentosum*, collected from several natural positions of Southeastern Poland, was carried out. Natural sites and abbreviations of the examined extracts are given in Table 1.

Oxygen radicals produced by human body cells, e.g., under the influence of pathogens, are the first line of defense against them. However, when radicals are produced for a long time, and in larger quantities, it can cause tissue damages and a chronic inflammation. Thus, antioxidant activity is partly responsible for the anti-inflammatory effect [53]. In traditional medicine, burdock root is used as an anti-inflammatory agent, e.g., in skin diseases [2,9]. Due to the above, our comparison of the two tested species began with the study of the ability of inhibition of lipoxygenase activity, the enzyme involved in the biosynthesis of pro-inflammatory leukotrienes, including LTB4, and antioxidant activity (scavenging of DPPH, O_2_^•−^, and H_2_O_2_).

## 2. Results and discussion

### 2.1. Evaluation of Lipoxygenase Activity Inhibition Ability in Cell-Free System

The ability to inhibit lipoxygenase activity by the tested extracts was not very high. Table 2 shows the inhibition of lipoxygenase activity by the extracts tested in the concentrations of 200 and 400 μg·mL^−1^. At a concentration of 400 μg·mL^−1^, extracts from the aerial parts and from the roots of *A. lappa* inhibit the enzyme activity by 28% and 32%, respectively, while extracts from the aerial parts and from the roots of *A. tomentosum* inhibit the enzyme activity by 23% and 25%, respectively. No statistically significant differences were found in the activity between *Arctium lappa* and *Arctium tomentosum* plant materials’ extracts. Moreover, there are no differences in activity between roots extracts and aerial parts extracts. Nordihydroguaiaretic acid, an IC_50_ (the concentration of the extract required to inhibit 50% of the enzyme activity) value of 127.04 ± 8.40 μg·mL^−1^, was used as a positive control. Studies carried out so far have shown that, as a raw material, burdock weakly inhibits lipoxygenase activity, with the IC_50_ value of 0.99 mg·mL^−1^ [54]. Moreover, Chagas-Paula et al. (2015) tested the ability to inhibit potato 5-lipoxygenase activity by 70% ethanolic extract from *Arctium lappa* leaves. The IC _50_ value for the tested extract was 17.6 µg·mL^−1^ [55]. So far, the effect of extracts of *Arctium tomentosum* raw materials on lipoxygenase activity has not been investigated.

### 2.2. Evaluation of ROS Scavenging in Cell-Free Systems

#### 2.2.1. Scavenging of DPPH

**Figure 1:**
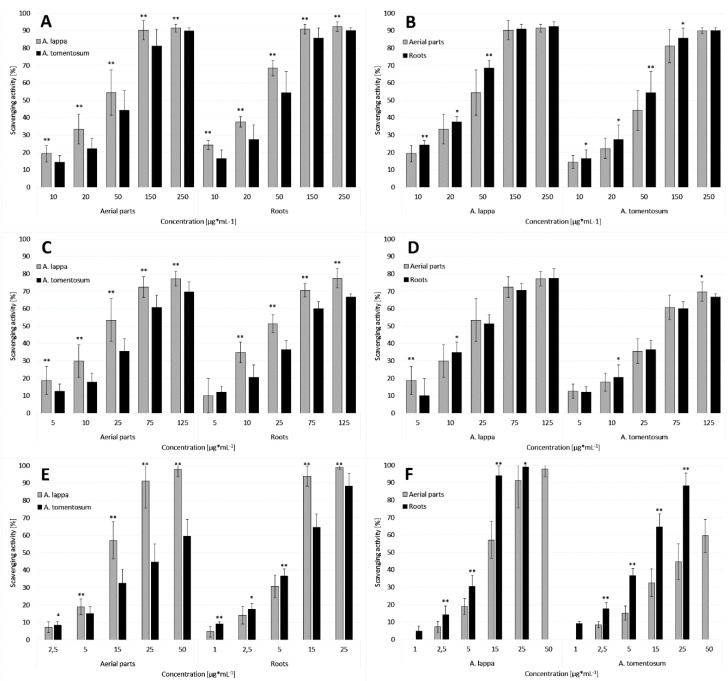
Average values of antioxidant activity of the extracts from the aerial parts and extracts from the roots of *Arctium lappa* and *Arctium tomentosum*. (**A**,**B**) DPPH scavenging activity, (**C**,**D**) O_2_^•−^ scavenging activity, and (**E**,**F**) H_2_O_2_ scavenging activity. Primes indicate statistically significant stronger activity of the particular extract at given concentration (* *p* < 0.05; ** *p* < 0.001).

In the evaluation of DPPH scavenging, statistically significant differences between the activity of extracts prepared from plant material collected from different natural sites were observed. The SC_50_ (extract concentration required to scavenge 50% of the radical) values against DPPH, presented in Table 2, were between 26 and 74 µg·mL^−1^. The ALRWA extract had the highest activity, while the weakest was ATAPC extract. Extracts from the aerial parts of *Arctium lappa* and from the roots of *Arctium lappa* were statistically significantly stronger than extracts from the aerial parts of *Arctium tomentosum* and from the roots of *Arctium tomentosum*, respectively (Figure 1A). The mean SC_50_ values were calculated, which were 37.55 ± 11.08 and 29.65 ± 4.03 μg·mL^−1^ for *A. lappa* aerial parts and for *A. lappa* roots, and 51.17 ± 13.33 and 42.67 ± 11.84 μg·mL^−1^ for *A. tomentosum* aerial parts and for *A. tomentosum* roots, respectively. It was observed that for both species, extracts prepared from roots had a stronger ability to scavenge DPPH (Figure 1B). Ascorbic acid, for which the SC_50_ value was 3.52 ± 0.26 μg·mL^−1^, was the positive control. According to the literature data, 50% ethanolic ultrasound-assisted leaf extract of *A. tomentosum* scavenged 62.88% DPPH [52], and aqueous *A. lappa* root extracts (32 mg) exhibit 80% scavenging activity against DPPH [22].

#### 2.2.2. Scavenging of the Superoxide Anion

The SC_50_ values of the scavenging capacity of superoxide anion by the tested extracts ranged from 15 to 75 μg·mL^−1^ (Table 2). The ALAPWA extract was the strongest against the O_2_^•−^, while the ATAPC extract was the weakest. The calculated mean SC_50_ values for extracts from the aerial parts and from the roots of *A. lappa* were 25.44 ± 10.73 and 27.50 ± 8.20 μg·mL^−1^, respectively, and for extracts from the aerial parts and from the roots of *A. tomentosum* were 45.94 ± 15.36 and 47.81 ± 10.36 μg·mL^−1^, respectively. Extracts prepared from plant materials from *A. lappa* were statistically significantly stronger in comparison to extracts from plant materials from *A. tomentosum* (Figure 1C). No statistically significant differences were observed in the scavenging capacity of superoxide anion between aerial parts extracts and roots extracts within the species (Figure 1D). Ascorbic acid with the SC_50_ value of 2.96 ± 0.24 μg·mL^−1^ was the positive control. The available literature also investigates that the superoxide radical anion scavenging ability increases with increasing extract content, and 60.5% O_2_^•−^ is scavenged by 1 mg of *A. lappa* aqueous root extract [22].

At the same time, to determine whether the activity in the system used is only a radical scavenging activity or also a xanthine oxidase inhibitory activity, the ability of the extracts to inhibit xanthine oxidase activity was measured. It was shown that the tested extracts do not significantly inhibit xanthine oxidase activity. Even at the highest concentration used, the enzyme inhibition did not exceed 9% for aerial parts extracts, and 18% for roots extracts. Allopurinol, whose IC_50_ value was 1.31 ± 0.16 μg·mL^−1^, was the positive control for inhibition of xanthine oxidase activity.

#### 2.2.3. Scavenging of Hydrogen Peroxide

The tested extracts have high scavenging activity against hydrogen peroxide (Table 2). The calculated mean SC_50_ values were 10.30 ± 2.35 and 5.68 ± 0.74 μg·mL^−1^ for the aerial parts and for the roots of *Arctium lappa*, respectively, and 33.74 ± 23.39 and 7.13 ± 1.58 μg·mL^−1^ for the aerial parts and for the roots of *Arctium tomentosum*, respectively. Extracts from *A. lappa* aerial parts, except for the lowest concentration used, scavenge hydrogen peroxide more strongly than extracts from *A. tomentosum* aerial parts (Figure 1E). At concentrations of 1–5 μg·mL^−1^, extracts from *A. tomentosum* roots had higher activity, whereas *A. lappa* roots extracts in concentrations of 15–25 μg·mL^−1^ (Figure 1E). Roots extracts have statistically significantly stronger activity than extracts from aerial parts (Figure 1F). Ascorbic acid, which was the positive control, used at a concentration of 1 μg·mL^−1^, scavenged almost 100% hydrogen peroxide. The conducted research confirmed the results previously published by Duh [22] that the extracts are capable to scavenge hydrogen peroxide in a concentration-dependent manner. According to Duh, 1 mg of *A. lappa* root aqueous extract scavenged 80.5% H_2_O_2_ [22].

**Figure 2:**
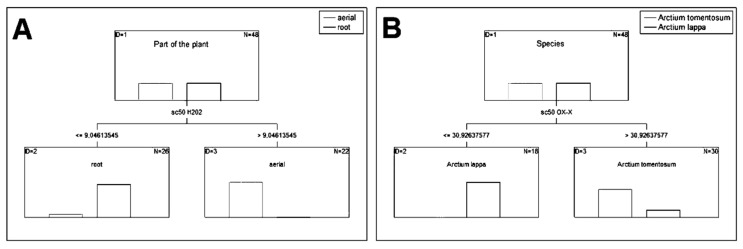
Classification and regression trees. (**A**): Classification and regression tree to distinguish plant parts based on SC_50_ value for scavenging of hydrogen peroxide; (**B**): Classification and regression tree to distinguish species based on SC_50_ value for scavenging of superoxide anion.

Based on the analyzed data, classification and regression trees (CART) were created. CART were used to learn how we can discriminate between the parts and species based on the antioxidant and anti-inflammatory activity. To distinguish between aerial parts and roots the best predictor is SC_50_ value for scavenging of hydrogen peroxide. The CART algorithm works as follows: If the parameter is greater than 9.05, then the respective extract was classified as obtained from the aerial parts otherwise from roots. This method achieved a 91.7% and 100% correct classification for aerial parts and for roots, respectively (Figure 2A). The best predictor to distinguish between species is SC_50_ value for scavenging of superoxide anion. If the parameter is greater than 30.9, then the extract belongs to the species *Arctium tomentosum*; otherwise, it belongs to *Arctium lappa*. The above rule allowed us to correctly classify 100% of the former species and 75% of the latter one (Figure 2B).

### 2.3. Phytochemical Analysis

#### 2.3.1. Total Content of Phenolic Compounds

The total content of phenolic compounds in the tested extracts is presented in Table 2. The calculated average contents of phenolic compounds in extracts from the aerial parts and from the roots of *A. lappa* were 113.01 ± 19.07 and 131.69 ± 14.74 mg·g^−1^, respectively, while in extracts from the aerial parts and from the roots of *A. tomentosum* were 78.52 ± 18.69 and 101.36 ± 28.28 mg·g^−1^, respectively. Extracts from *A. lappa* contained a statistically significant higher content of phenolic compounds than extracts from *A. tomentosum*. Roots extracts contained more phenolic compounds, as compared to extracts from the aerial parts. The obtained results are comparable with the available literature data. According to Lee et al. [28] the total phenolic content in the 70% ethanolic extract of *A. lappa* leaves is 97.49 mg·g^−1^, whereas Haghi et al. [17] investigated that he roots of a cultivated greater burdock had a higher total phenolic content than the leaves (137 and 41.4 mg in 100 g dry material, respectively). Meanwhile, the total polyphenolic content in the 50% ethanolic leaf extract of *A. tomentosum* prepared under reflux is 55 mg·g^−1^ [52].

**Figure 3:**
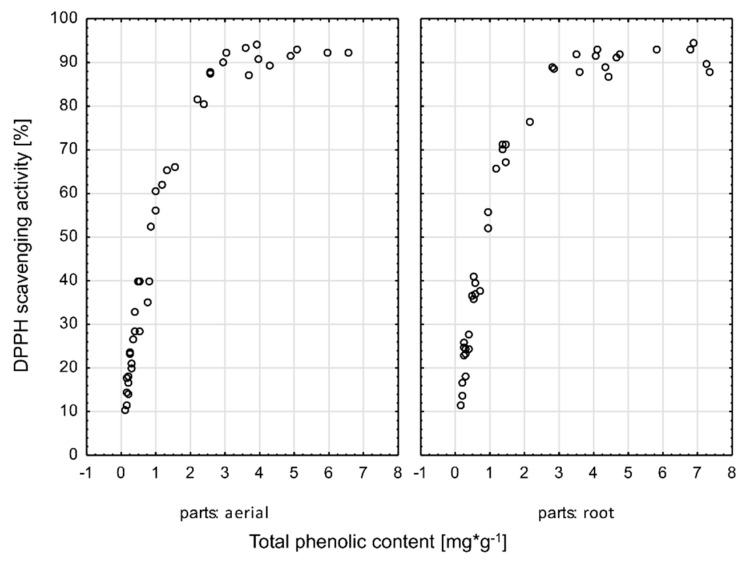
Correlation between the ability to scavenge DPPH and the content of polyphenolic compounds in the tested extracts.

A correlation between antioxidant activity and the content of polyphenolic compounds in the tested extracts was observed. All tested samples showed activity in a concentration-dependent manner. As an example, the correlation between the ability to scavenge DPPH and the content of polyphenolic compounds in the extract tested was shown. The average Spearman correlation between the scavenging activity of DPPH and content of phenolic compounds is statistically significant, positive, and strong, with r = 0.965. Scatterplots correlation for aerial parts (r = 0.973) and for roots (r = 0.952) are presented on Figure 3. The scatter diagrams are similar for two species (*Arctium tomentosum* with r = 0.989 and *Arctium lappa* with r = 0.939) and places—the respective Spearman’s rank correlation coefficient for different places is between 0.915 and 0.989.

#### 2.3.2. HPLC–DAD–MS^n^

**Figure 4:**
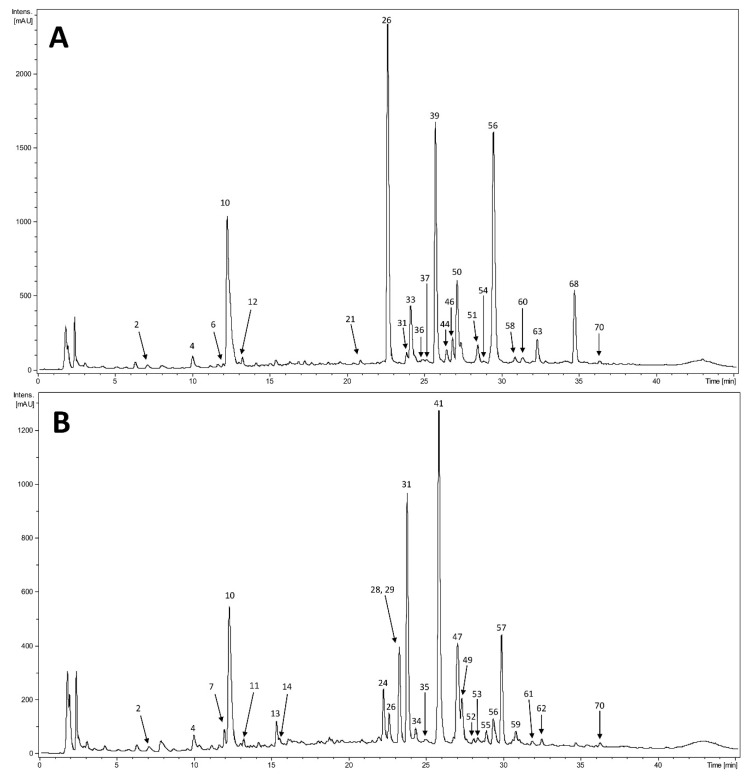
HPLC chromatograms at 254 nm of extracts from aerial parts of: (**A**) *Arctium lappa*, plant material collected from the natural position of Wola Wyżna A; (**B**) *Arctium tomentosum*, plant material collected from the natural position of Strzyżów, Zadworze Street.

**Figure 5:**
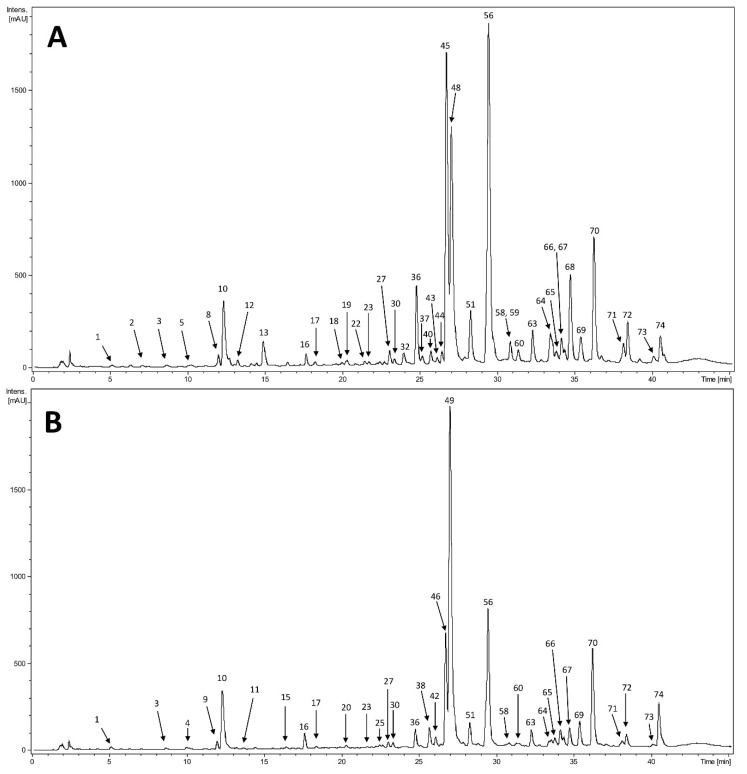
HPLC chromatograms at 254 nm of extracts from roots of: (**A**) *Arctium lappa*, plant material collected from the natural position of Wola Wyżna A; (**B**) *Arctium tomentosum*, plant material collected from the natural position of Strzyżów, Zadworze Street.

HPLC–DAD–MS^n^ analysis of all sixteen extracts was carried out. Chromatograms and results of selected samples were presented: ALAPWA (Figure 4A), ATAPZ (Figure 4B), ALRWA (Figure 5A), and ATRZ (Figure 5B). The results are shown in Table 3. Almost all compounds present in roots extracts and many compounds contained in extracts from aerial parts showed maxima UV at approximately 240, 300, and 310–325 nm. Additionally, the shape of recorded spectra was characteristic for phenolic acids, especially caffeic acid derivatives. In addition, in the extracts from the aerial parts compounds that displayed absorption maxima at ca. 250–265 nm and ca. 330–360 nm were observed. These compounds were preliminarily assigned to flavonoids based on their UV–Vis spectra. Further identification was performed based on MS spectra in negative ion mode.

Compounds **2**, **10**, and **11** with pseudomolecular ion at *m/z* 353, fragmenting in MS^2^ to ions at *m/z* 191 and *m/z* 179 or *m/z* 173 were identified as isomers of caffeoylquinic acid based on the hierarchical key created by Clifford [56]. Compounds, which in MS^2^ fragmented to the base ion at *m/z* 515, and then to ions at *m/z* 353 and *m/z* 191, were identified as dicaffeoylquinic acid derivatives [57]. These compounds predominated in the tested extracts. Compound **30** showing base peak ion at *m/z* 677, fragmenting in MS^2^ to the base peak at *m/z* 515, and then in MS^3^ to the base peak at *m/z* 353 was identified as tricaffeoylquinic acid isomer, but its further assignment was not possible, due to the lack of proper texts from the literature. The other compounds, with the UV–Vis spectrum characteristic of phenolic acids (maxima at 240 and 325 nm), were tentatively identified on the basis of comparisons of fragmentation spectra with previous reports [18,20] as derivatives of caffeoylquinic acid containing caffeic acid and/or aliphatic acid substituents as ester groups. Loss of characteristic neutral residues was observed in MS^2^ spectra. The cleavage of a fragment with mass 98 amu corresponded to the cleavage of fumaroyl moiety, loss of 100 amu corresponded to cleavage of the succinoyl moiety, and loss of 116 amu corresponded to cleavage of the maloyl moiety.

Compounds showing pseudomolecular ions at *m/z* 613 (**5**, **16**, **38**, **40**, **42**, and **50**), *m/z* 615 (**56**) and *m/z* 631 (**36**, **44**, **46**, **48**, and **52**), fragmenting in MS^2^ to base peaks at *m/z* 515, and then in MS^3^ to base peaks at *m/z* 353, were identified as isomers of dicaffeoylfumaroylquinic acid, dicaffeoylsuccinoylquinic acid, and dicaffeoylmaloylquinic acid, respectively. Compound **68** with the parent ion at *m/z* 715, cleaving succinoyl moiety (-100 amu) or caffeoyl moiety (-162 amu) in the fragmentation spectrum, was classified as dicaffeoyldissuccinoylquinic acid isomer. Compounds **32**, **45**, and **51** with the parent ion at *m/z* 747, fragmenting with the loss of maloyl moiety (-116 amu) or caffeoyl moiety (-162 amu), were classified as dicaffeoyldimaloylquinic acid isomers. Compounds with the base peak ion at *m/z* 713 (**63**) or at *m/z* 731 (**58**) that cleaved 98 amu (fumaroyl residue), 100 amu (succinoyl residue), 116 amu (maloyl residue), or 162 amu (caffeoyl residue) as a result of fragmentation were identified as isomers of diccaffeoylfumaroylsuccinoylquinic acid and dicaffeoylmaloylsuccinoylquinic acid, respectively. Compound **17** that displayed pseudomolecular ion at m/z 367 with base peak ion in fragmentation spectrum at m/z 191 was assigned as 5-*O*-feruoylquinic acid according to Clifford et al. [56].

Compounds with UV–Vis maxima at about 250–265 nm and 340–360 nm were present in extracts from aerial parts. They were initially classified as flavonoids. Compounds **33**, **34**, **39**, **47**, and **57** characterized by UV–Vis maxima at about 265 and 344 nm and having aglycone residue ion at *m/z* 285 in the MS^2^ fragmentation spectrum were assigned kaempferol glycosides. Compounds **24**, **26**, **28**, **31**, **41**, and **55** having UV–Vis maxima at about 250 and 350 nm and fragmenting in the MS^2^ spectrum to a characteristic base peak at *m/z* 301 were classified as quercetin derivatives. Compounds **28**, **31**, and **47** cleaving in the MS^2^ spectrum of glucosyl or galactosyl moiety with neutral loss of 162 amu were identified as flavonoid hexosides. Compounds **33** and **54** showing neutral loss of 176 amu (corresponding to uronic acid) were characterized as flavonoid glucuronides. Cleavage of 308 amu, with a characteristic neutral loss of 162 amu (corresponding to hexose) or 146 amu (typical for the cleavage of rhamnose moiety), allowed classification of compounds **26**, **34**, and **39** as flavonoid rhamnohexosides. Compounds **41**, **55**, and **57** showing 248 amu loss and in their fragmentation spectrum a characteristic ion corresponding to decarboxylation (-44 amu) was present, were classified as flavonoid malonylhexosides. 

The aerial parts of *Arctium lappa* are rich in phenolic acids, primarily derivatives of dicaffeoylquinic acid isomers with fumaric, succinic, and malic acid residues in the side chain. Flavonoids are also found in the extracts: quercetin and kaempferol derivatives. The most intense peaks correspond to chlorogenic acid, quercetin rhamnohexoside (rutin), kaempferol rhamnohexoside, and dicaffeoylsuccinoylquinic acid. In the aerial parts of *Arctium tomentosum*, derivatives of dicaffeoylquinic acid isomers are also present. Among flavonoids it was kaempferol and quercetin derivatives that predominated in the analyzed extracts. The most intense peaks corresponded to chlorogenic acid, hexoside and malonylhexoside of quercetin, and hexoside and malonylhexoside of kaempferol. The most abundant peaks found in both species’ roots were chlorogenic acid, dicaffeoylmaloylquinic acid, and dicaffeoylsuccinoylquinic acid.

## 3. Materials and Methods

### 3.1. Chemicals

Allopurinol, apigenin 7-*O*-glucuronide, ascorbic acid, chlorogenic acid, 2,2-diphenyl-1-picrylhydrazyl (DPPH), horseradish peroxidase (HRP), hydrogen peroxide (H_2_O_2_), kaempferol 3-*O*-glucuronide, linoleic acid, lipoxygenase from Glycine max, luminol, nitrotetrazolium blue chloride (NBT), nordihydroguaiaretic acid, quercetin, quercetin 3-*O*-galactoside (hyperoside), quercetin 3-*O*-glucoside (isoquercitrin), xanthine, and xanthine oxidase were purchased from Sigma-Aldrich Chemie GmbH (Steinheim, Germany). Boric acid, ethanol, Folin–Ciocalteu reagent, sodium carbonate and sodium hydroxide were purchased from Avantor Performance Materials POCH (Gliwice, Poland). Gallic acid was purchased from ROTH (Karlsruhe, Germany). Phosphate-buffered saline (PBS) was purchased from Biomed (Lublin, Poland). Water was obtained by using Milli-Q Plus, MILLIPORE (Billerica, MA, USA) (18.2 MΩ cm).

### 3.2. Plant Material and Extracts Preparation

Plant material was harvested at the turn of June and July 2016, from eight natural sites of the southeast region of Poland (near Rzeszów). The aerial parts and roots of *Arctium lappa* were collected in Jaszczurowa, Wola Wyżna, and Jaśliska. The aerial parts and roots of *Arctium tomentosum* were collected in Czudec, Kołaczyce, and Strzyżów. The geographical coordinates are given in Table 1.

The plant material was authenticated by Dr Maria Ziaja, according to “*A key for identification of vascular plants of Lowland Poland”* [47]. Specimen of raw materials (Table 1) are available at the Department of Pharmacognosy and Molecular Basis of Phytotherapy, Medical University of Warsaw, Warsaw, Poland. Raw materials were dried at room temperature, in the shade. 

The obtained plant materials were ground with an IKA MZO electric grinder (IKA-WERKE, Staufen im Breisgau, Germany), and then 70% (*v/v*) ethanolic extracts were prepared. A three-time extraction was carried out, under reflux, at 100 °C, for 1 hour each time, using 200 mL 70 % (*v/v*) ethanol for 10.0 g powdered plant material. The obtained extracts were filtered through cotton and through a paper filter (389Ø). Next, the organic solvent was evaporated under the vacuum (LABORANTA 4000 WB Heidolph), at 45 °C. In the case of concentrated, ethanol-free extracts from the aerial parts, an additional step was performed—purification with chloroform to remove the chlorophyll. A three-time liquid/liquid extraction was performed each time, using 200 mL of chloroform. After the extraction, the chloroform residue was evaporated from the aqueous layer, using a rotary vacuum evaporator at 45 °C. The concentrated aqueous extracts were frozen to -72 °C and then lyophilized by using a laboratory freeze-dryer Cryodos (Telsar, Terrassa, Spain). The dry residues were homogenized in a mortar, weighed, and placed in sealed vials. The abbreviations and masses of powdered plant material and obtained extracts are given in Table 1. They were stored at 2–8 °C.

### 3.3. Evaluation of Lipoxygenase Activity Inhibition Ability in Cell-Free System

Inhibition of lipoxygenase (LOX) activity was determined by the method according to SIGMA Enzymatic Assay of Lipoxygenase (EC 1.13.11.12), which was modified to 96-well microliter plates’ volume (final sample volume 200 µL) [58]. Then, 50 µL of extracts dissolved in borate buffer (200 mM, pH = 9.0 at 25 °C) was mixed with 100 µL of linoleic acid (LA) solution (322.5 μM LA in the final sample volume) and 50 μL of LOX solution in borate buffer (315.45 U*mL^-1^ in the final sample volume). The study was performed on transparent 96-well plates without self-absorption. The measurement of the absorbance at 234 nm was done after 7 minutes of incubation, at room temperature, in the absence of light. The percentage of LOX inhibition was calculated in comparison to the control, without test extracts. Nordihydroguaiaretic acid was used as a positive control.

### 3.4. Evaluation of ROS Scavenging in Cell-Free Systems

#### 3.4.1. Scavenging of DPPH

Scavenging of DPPH (2,2-diphenyl-1-picrylhydrazyl) was examined by using the method of Choi et al. [59]. Then, 100 µL of extract solutions in 50% (*v/v*) ethanol, at concentrations of 10, 20, 50, 150, and 250 µg·mL^−1^, was mixed in a 96-well plate with 100 µL of a 0.02 mM solution of DPPH dissolved in 99.8% (*v/v*) ethanol. After 30 minutes of incubation in the dark, at room temperature, absorbance at 518 nm was measured in a Synergy 4 microplate reader (BioTek, Winooski, USA). The scavenging rate of DPPH was calculated relative to a control without the tested extracts. Ascorbic acid was used as a positive control.

#### 3.4.2. Scavenging of the Superoxide Anion

Scavenging of the superoxide anion (O_2_^•−^) was examined by using a xanthine–xanthine oxidase system with the NBT (nitro blue tetrazolium chloride) reduction method as described by Choi et al. [59]. Then, 50 µL of extract, dissolved in PBS, at concentrations of 5, 10, 25, 75, and 125 μg·mL^−1^, was mixed in a 96-well plate with 100 µL of a mixture of xanthine with NBT (1:1 (*v/v*); 0.4 mM xanthine and 0.24 mM NBT in PBS) and 50 µL of a solution of xanthine oxidase in PBS (prepared ex tempore, 3.66 mU of xanthine oxidase in PBS). The absorbance at 560 nm was measured in a Synergy 4 microplate reader (BioTek, Winooski, USA) after 20 minutes of the plate incubation, at 37 °C, in the absence of light. The percent of inhibition of the xanthine/xanthine oxidase system was calculated in comparison to the control without tested extracts. Ascorbic acid was a positive control.

To evaluate whether extracts affected the superoxide anion generation by direct interaction with xanthine oxidase, the enzyme activity was determined by monitoring the uric acid formation [60]. Then, 50 μL of the extract, dissolved in PBS at concentrations of 5, 10, 25, 75, and 125 μg·mL^−1^, was mixed in a 96-well plate with 100 μL of xanthine solution (0.4 mM in PBS) and 50 µL of xanthine oxidase (prepared ex tempore, 3.66 mU in PBS). The absorbance at 285 nm was measured in a Synergy 4 microplate reader (BioTek, Winooski, USA) after 20 minutes of a plate incubation at 37 °C, in the absence of light. The percentage of xanthine oxidase activity was calculated in comparison to the control without test extracts. Allopurinol was used as a positive control.

#### 3.4.3. Scavenging of Hydrogen Peroxide

Scavenging of hydrogen peroxide (H_2_O_2_) was performed with horseradish peroxidase, as described by O’Dowd et al. [61]. Then, 50 µL of the extract in PBS, at concentrations of 2.5, 5, 15, 25, and 50 µg·mL^−1^ for aerial parts extracts and 1, 2.5, 5, 15, and 25 µg·mL^−1^ for roots extracts, was mixed in a white 96-well plate with 50 µL of horseradish peroxidase (solution in PBS, prepared ex tempore, 98.8 mU HRP), 50 µL hydrogen peroxide (solution in PBS, prepared ex tempore, 0.0075 % H_2_O_2_), and 50 µL of luminol (0.005 mg·mL^−1^ in PBS). The chemiluminescence was measured in a Synergy 4 microplate reader (BioTek, Winooski, USA), at room temperature, in the absence of light, 5 minutes after the addition of the luminol solution. The reader was set to read luminescence at sensitivity 75. The percent of inhibition of the HRP/hydrogen peroxide system was calculated in comparison to the control without test extracts. Ascorbic acid was used as a positive control.

### 3.5. Phytochemical Analysis

#### 3.5.1. Total Content of Phenolic Compounds

Determination of the total phenolic compounds was carried out by colorimetric method with the Folin–Ciocalteu reagent on a 96-well plate. In total, 40 µL of the tested extract at concentration 1 mg·mL^−1^ dissolved in 50 % (*v/v*) methanol was mixed with 105 µL of a 10 % (*v/v*) of Folin–Ciocalteu reagent and 85 µL of 1 M sodium carbonate solution. The mixture was incubated for 15 minutes at 45°C on a microplate shaker (DTS-2, Elmi) that allowed the samples to be mixed simultaneously (at 420 RMP). Then the absorbance, at 765 nm, was measured. The content of polyphenols in the tested extracts was calculated to gallic acid, for which a calibration curve was prepared.

#### 3.5.2. HPLC–DAD–MS^n^

The HPLC–DAD–MS^n^ analysis was performed by using an UltiMate HPLC 3000 system (Dionex, Germany) with DAD detection and splitless connection with an AmaZon SL ion trap mass spectrometer with an ESI interface (Bruker Daltonik, GmbH, Germany). The concentration of the tested samples was 5 mg·mL^−1^ and the injection volume was 5 μL. HPLC analysis was carried out on a reversed-phase Zorbax SB C18, 150 mm × 2.1 mm, 1.9 μm column (Agilent, CA, USA). The column oven temperature was set to 25 °C. The mobile phase (A) was water/formic acid (100:0.1, *v/v*), and the mobile phase (B) was acetonitrile/formic acid (100:0.1, *v/v*). The flow rate was 0.2 mL·min^−1^. The gradient system was 0–10 min 7–15% B, 10–35 min 15–30% B, and 35–45 min 30–95% B. The column was equilibrated for 10 min between injections. UV–Vis spectra were recorded over a range of 200–450 nm, and chromatograms were acquired at 254, 280, 325, and 350 nm. The elute was introduced directly into the ESI interface. The nebulizer pressure was 40 psi, dry gas flow was 9 L·min^−1^, dry temperature was 300 °C, and the capillary voltage was 4.5 kV. The MS spectra were registered by scanning from *m/z* 70 to 2200. Compounds were analyzed in a negative ion mode. The MS^2^ fragmentation was obtained for two of the most abundant ions at the time. Identification of compounds was performed based on the literature data [18,57,62].

### 3.6. Statistical Analysis

For each assay, three independent experiments were performed in triplicate. To characterize the considered parameters, mean and standard deviation were computed. Data were analyzed by using Statistica (data analysis software system), version 13 (TIBCO Software Inc., 2017). The normal distribution was checked by the Shapiro–Wilk test, and the homogeneity of variance by the Brown and Forsyth test. Statistical significance was determined by one-way ANOVA, with Dunnett’s test and post hoc Tuckey’s test, or Kruskal–Wallis test. To create a predictive model, CART analysis was applied. The purpose of this analysis was to learn how one can discriminate between the two species, based on the tested parameters. Each independent variable was examined, and a split was made to maximize the sensitivity and specificity of the classification, resulting in the development of a decision tree. To assess the strength of a relationship between two variables/parameters, the Pearson correlation coefficient was used when the relation was linear and both variables were normally distributed; otherwise, Spearman’s rank coefficient was applied. All computations were applied at a significance level of 0.05.

## 4. Conclusions

The conducted research shows too many significant differences between the extracts of *Arctium lappa* and *Arctium tomentosum* for these two species to be considered as providing equivalent plant material. They differ not only in the content of phenolic compounds and antioxidant activity, but also in their chemical composition. To the best of our knowledge, there is no available texts in the literature that provide data on the comparison of the anti-lipoxygenase and the antioxidant activity, as well as the chemical composition between raw materials obtained from the two tested species. Due to the obtained results and the very small number of research documenting both the chemical composition and activity of plant materials obtained from the species *Arctium tomentosum*, in our opinion, it is not justified to include this species as a source equal to *Arctium lappa* for obtaining *Arctii radix* (*Bardanae radix*) in the monography of European Medicine Agency [48].

Moreover, statistically significant differences in the activity and content of phenolic compounds were also observed between extracts made from a specific part of a plant of the same species but collected from other natural sites. Despite the fact that plant material was collected from natural sites not very distant from each other (around Rzeszów, the region of Southeastern Poland), the differences were significant. This draws attention to the need to standardize extracts that would be used in medicine, to the content of the main active compounds.

## Figures and Tables

**Table 1:** Natural sites, geographical coordinates, specimens of raw material, abbreviations, and masses of powdered plant material and prepared 70% (*v/v*) ethanolic extracts.

Species	Natural Site	Geographical Coordinates	Specimens of Raw Material	Part of the Plant	Abbreviation	Mass of Plant Material (g)	Mass of Lyophilized Extracts (g)
*Arctium lappa*	Jaszczurowa	49°53′03″ N; 21°33′40″ E	AL/16/J	Aerial parts	ALAPJ	30.00	6.69
				Roots	ALRJ	30.00	4.45
	Wola Wyżna A	49°23′30″ N; 21°52′24″ E	AL/17/WA	Aerial parts	ALAPWA	36.70	2.24
				Roots	ALRWA	19.28	2.65
	Wola Wyżna B	49°23′30″ N; 21°52′24″ E	AL/17/WB	Aerial parts	ALAPWB	30.00	2.23
				Roots	ALRWB	14.10	2.95
	Jaśliska	49°23′38″ N; 21°52′45″ E	AL/17/ZJ	Aerial parts	ALAPZJ	28.90	2.30
				Roots	ALRZJ	13.70	2.55
*Arctium tomentosum*	Czudec	49°56′44″ N; 21°50′17″ E	AT/16/C	Aerial parts	ATAPC	30.00	5.96
				Roots	ATRC	30.00	4.06
	Kołaczyce	49°48′30″ N; 21°26′25″ E	AT/16/K	Aerial parts	ATAPK	23.90	4.35
				Roots	ATRK	17.77	2.75
	Strzyżów	49°52′15″ N; 21°47′28″ E	AT/16/S	Aerial parts	ATAPS	16.90	3.64
				Roots	ATRS	28.83	3.88
	Strzyżów, Zadworze st.	49°52′15″ N; 21°47′28″ E	AT/16/Z	Aerial parts	ATAPZ	26.80	5.40
				Roots	ATRZ	30.00	6.94

**Table 2:** Inhibition of lipoxygenase (LOX) activity, SC_50_ (extract concentration required to scavenge 50% of the radical) values of scavenging of DPPH, superoxide anion, and hydrogen peroxide, as well as total phenolic content in the tested extracts.

Species	Sample	LOX Inhibition ± SD (%)		SC_50_ ± SD (μg·mL^−1^)			Total Phenolic Content ± SD (mg·g^−1^)
		200 μg·mL^−1^	400 μg·mL^−1^	DPPH	O_2_^•−^	H_2_O_2_	
***Arctium lappa***	ALAPJ	8.85 ± 0.84	22.58 ± 1.38	56.70 ± 3.40	42.44 ± 2.47	15.79 ± 1.11	74.15 ± 7.49
	ALAPWA	11.46 ± 2.04	38.45 ± 1.69	29.28 ± 2.97	26.00 ± 4.70	8.68 ± 0.37	131.14 ± 9.35
	ALAPWB	7.80 ± 2.19	24.58 ± 1.49	36.48 ± 3.22	15.42 ± 3.16	9.53 ± 0.45	101.67 ± 7.27
	ALAPZJ	7.12 ± 2.59	28.87 ± 2.45	30.48 ± 3.56	20.41 ± 4.12	10.62 ± 0.62	119.16 ± 7.29
	ALRJ	9.53 ± 0.94	41.15 ± 1.49	31.96 ± 2.56	29.82 ± 2.57	5.12 ± 0.18	147.14 ± 11.88
	ALRWA	11.42 ± 1.89	31.09 ± 2.78	26.78 ± 3.19	29.57 ± 4.13	5.66 ± 0.29	137.43 ± 9.74
	ALRWB	7.16 ± 2.01	22.71 ± 2.15	31.14 ± 3.02	20.17 ± 3.79	6.32 ± 0.37	116.61 ± 9.90
	ALRZJ	8.87 ± 2.47	31.27 ± 2.61	29.03 ± 3.38	30.25 ± 4.50	5.54 ± 0.28	135.87 ± 11.46
***Arctium tomentosum***	ATAPC	5.42 ± 1.10	13.12 ± 0.85	74.19 ± 4.38	75.88 ± 6.37	80.36 ± 6.97	51.55 ± 7.05
	ATAPK	9.74 ± 1.09	23.39 ± 2.38	43.28 ± 1.85	35.56 ± 2.47	28.64 ± 2.13	85.61 ± 9.28
	ATAPS	15.77 ± 1.12	36.18 ± 1.34	38.48 ± 2.37	36.21 ± 2.71	18.07 ± 1.31	97.84 ± 8.10
	ATAPZ	11.49 ± 1.08	21.20 ± 1.09	54.87 ± 2.80	47.68 ± 5.86	45.15 ± 5.52	79.09 ± 6.30
	ATRC	8.49 ± 0.74	25.85 ± 1.72	40.70 ± 2.64	49.11 ± 6.39	5.83 ± 0.20	95.45 ± 9.06
	ATRK	9.90 ± 0.83	38.47 ± 2.49	28.70 ± 2.93	37.26 ± 6.08	6.97 ± 0.61	145.09 ± 11.69
	ATRS	8.53 ± 0.62	16.41 ± 0.83	59.94 ± 3.94	58.91 ± 4.32	8.38 ± 0.66	71.74 ± 4.14
	ATRZ	7.56 ± 0.77	20.54 ± 1.40	44.62 ± 2.08	46.70 ± 5.08	8.10 ± 0.48	93.16 ± 6.68

**Table 3:** UV–Vis and MS data of major compounds found in aerial parts and in roots of *Arctium lappa* and *Arctium tomentosum.*

No.	Compound	Rt (min)	UV–Vis maxima (nm)	[M-H]^-^ *m/z*	MS^2^ ions	MS^3^ ions	NL (amu)	AL- APWA	ATAPZ	ALRWA	ATRZ
**1**	caffeoylquinic acid derivative	5.2	240, 301sh, 324	451	**353b**, 191	191b, 179				+	+
**2**	3-*O*-caffeoylquinic acid * (neochlorogenic acid)	7.2	305sh, 321	353	**191b**, 179	191	162	+	+	+	
**3**	caffeoylfumaroylmaloylquinic acid	8.8	241sh, 305sh, 327	567	**469b**, 387, 307	353, 307b, 277, 191	162			+	+
**4**	caffeoylquinic acid dimer	10.1	248sh, 286, 328	705	595, 513b, 339, 229			+	+		+
**5**	dicaffeoylfumaroylquinic acid	10.3	300sh, 315	613	**515b**, 409	353, 323b, 191, 179	162			+	
**6**	undefined compound	11.8	217, 265	643	**545b**, 203	341b, 203		+			
**7**	undefined compound	11.9	217, 265	537	**375b**, 345, 327	345b, 327	162		+		
**8**	undefined compound	12.0	217, 265	643	**545b**, 203	341b, 203				+	
**9**	undefined compound	12.0	218, 265	503	**341b**, 281, 179	281b, 251, 179	162				+
**10**	5-*O*-caffeoylquinic acid ^#^ (chlorogenic acid)	12.4	235, 306sh, 320	353	**191b**, 179	191	162	+	+	+	+
**11**	4-*O*-caffeoylquinic acid * (cryptochlorogenic acid)	13.3	306sh, 323	353	**191**, 179, 173b, 135	191	162		+		+
**12**	4-*O*-caffeoylquinic acid derivative	13.4	306sh, 324	593	**353**, 239b	191, 179, 173b, 155	162	+		+	
**13**	undefined compound	15.3	280, 308sh	433	221b, 177				+	+	
**14**	undefined compound	15.3	280, 308sh	485	467, 399, 305b, 189				+		
**15**	5-*O*-*p*-coumaroylquinic acid *	16.4	301sh, 311	337	**191b**, 163	191	146				+
**16**	dicaffeoylfumaroylquinic acid	17.7	239sh, 301sh, 320	613	**515b**	353b, 335, 191, 179	162			+	+
**17**	5-*O*-feruloylquinic acid *	18.2	297	367	**191b**, 173	191	176			+	
**18**	caffeoylquinic acid derivative	20	300sh, 317	649	533, **487b**, 451, 371, 353, 335	371b, 353, 289, 191	162			+	
**19**	dicaffeoylquinic acid derivative	20.1	300sh, 317	955	839, 613, 515b, 341					+	
**20**	tricaffeoylquinic acid derivative	20.4	302sh, 319	839	**677**, 647, 515b, 323	515b, 485, 323	162				+
**21**	caffeoylquinic acid derivative	20.9	308sh, 327	651	**553b**	453, 391, 353b, 291, 191		+			
**22**	dicaffeoylmaloylquinic acid derivative	21.5	234, 303sh, 321	793	**631**, 613, 515b, 497, 341	515b, 341	162			+	
**23**	caffeoylmaloylquinic acid	21.7	232, 303sh, 319	469	419, 388, 323					+	+
**24**	quercetin derivative	22.3	255, 352	609	343, 301b, 271				+		
**25**	undefined compound	22.6	303sh, 321	431	351, 263b, 247, 121						+
**26**	quercetin rhamnohexoside	22.8	255, 353	609	343, 301b, 255			+	+		
**27**	tricaffeoylquinic acid derivative	23.1	234, 303sh, 321	793	**677**, **631b**, 515, 469, 353	(677) 515b, 353, 335				+	+
						(631) 515b, 439, 341	162				
**28**	quercetin 3-*O*-galactoside (hyperoside) ^#^	23.3	255, 352	463	343, 301b, 179				+		
**29**	apigenin derivative	23.4	255, 264sh, 352, 374sh	449	269b, 225, 207				+		
**30**	tricaffeoylquinic acid	23.4	301sh, 321	677	**515b**, 485	353, 323b, 191	162			+	+
**31**	quercetin 3-*O*-glucoside (isoquercitrin) ^#^	23.9	251, 305sh, 333	463	301b, 257, 179			+	+		
**32**	dicaffeoyldimaloylquinic acid	24	235sh, 302sh, 326	747	**631b**, 469	515b, 469, 353, 335				+	
**33**	kaempferol 3-*O*-glucuronide ^#^	24.2	253, 343	461	357, 285b			+			
**34**	kaempferol rhamnohexoside	24.4	265, 331	593	**447**, 327, 285b	357, 285b	146		+		
**35**	undefined compound	24.7	265, 331	701	655, 509, 335b, 263				+		
**36**	dicaffeoylmaloylquinic acid	24.9	238, 304sh, 326	631	515, **469b**, 353	353b, 173	162	+		+	+
**37**	tricaffeoylsuccinoylquinic acid	25.3	296, 323	777	677, **615b**, 515	515b, 453, 353	162	+		+	
**38**	dicaffeoylfumaroylquinic acid	25.7	238sh, 305sh, 325	613	**515b**, 433	353b, 299, 203	162				+
**39**	kaempferol rhamnohexoside	25.8	264, 343	593	285			+			
**40**	dicaffeoylfumaroylquinic acid	25.8	237sh, 305sh, 325	613	**515b**, 433, 353	353b, 299, 203				+	
**41**	quercetin malonylhexoside	25.9	256, 353	549	505b, 463, 301				+		
**42**	dicaffeoylfumaroylquinic acid	26.1	236sh, 305sh, 325	613	**515b**	353b, 335, 203, 191, 173	162				+
**43**	dicaffeoylcoumaroylmaloylquinic acid	26.2	242sh, 305sh, 324	777	**631**, **615**, **515b**	(631) 515	146			+	
						(615) 515, 453, 353b	162				
						(515) 353, 191, 179, 173b	162				
**44**	dicaffeoylmaloylquinic acid	26.5	239sh, 306sh, 327	631	**469b**, 353, 191	353, 191, 173		+		+	
**45**	dicaffeoyldimaloylquinic acid	26.7	241, 308, 332, 351	747	**631b**, **585**, 469	(631) 469b, 353	162			+	
						(585) 469b, 353	162				
**46**	dicaffeoylmaloylquinic acid	26.9	238sh, 306sh, 328	631	**469b**, 353, 191	353b, 307, 191	162	+			+
**47**	kaempferol hexoside	27.1	240sh, 265, 303sh, 328	447	327, 285b, 255				+		
**48**	dicaffeoylmaloylquinic acid	27.2	235, 303sh, 327	631	**515**, **469b**, 353, 307, 191	(515) 353b, 335, 191	162			+	
						(469) 353b, 307, 191	162				
**49**	dicaffeoylquinic acid derivative	27.2	237, 303sh, 327	767	**515b**, 353	353b, 335, 191	162		+		+
**50**	dicaffeoylfumaroylquinic acid	27.4	234sh, 304sh, 326	613	**515b**, 353	353b, 191	162	+			
**51**	dicaffeoyldimaloylquinic acid	28.3	237, 306sh, 328	747	**631**, **585**, 469b, 353	(631) 469b, 353	162	+		+	+
						(585) 469b, 353					
**52**	dicaffeoylmaloylquinic acid	28.4	266, 282, 327	631	**469b**, 451, 353, 335	353b, 307, 191	162		+		
**53**	undefined compound	28.4	266, 282, 327	417	327, 284b, **255**	255	162		+		
**54**	apigenin 7-*O*-glucuronide ^#^	28.5	266, 331	445	269b, 175			+			
**55**	quercetin malonylhexoside	29	253, 335	549	**505b**	463, 445, 301b			+		
**56**	dicaffeoylsuccinoylquinic acid	29.6	241, 306sh, 327	615	**515**, **453b**, 353, 191	(515) 353b, 335, 299, 255, 203, 173	162	+	+	+	+
						(453) 353	162				
**57**	kaempferol malonylhexoside	29.9	264, 343	533	**489b**, 285	285b, 255			+		
**58**	dicaffeoylmaloylsuccinoylquinic acid	30.9	237sh, 310sh, 328	731	**569**, 469b, 451, 353	489, 469b, 353, 289	162	+		+	+
**59**	caffeic acid derivative	31	215, 245sh, 306sh, 328	459	**297b**, 179, 135	279, 179, 135b	162		+	+	
**60**	coumaroylcaffeoylquinic acid	31.4	304sh, 327	499	455, 353b, 337, 191			+		+	+
**61**	caffeic acid derivative	31.9	210, 324	557	**459b**, 297	297b, 179, 135			+		
**62**	undefined compound	32.5	266, 316	533	485b, 352, 315, 293				+		
**63**	dicaffeoylsuccinoylfumaroylquinic acid	32.4	236sh, 307sh, 328	713	**615b**, 453	515, 453, 353b, 191		+		+	+
**64**	tricaffeoylquinic acid derivative	33.5	308sh, 325	909	793b, **677**, 613	515, 497b, 469, 353	162			+	+
**65**	dicaffeoylmaloylquinic acid derivative	33.9	232sh, 307sh, 326	793	**631b**, 613	515, 469b, 451, 353	162			+	+
**66**	dicaffeoylmaloylquinic acid derivative	34.2	241sh, 308sh, 325	793	**631b**, 613, 497, 469	515, 469b, 451, 353	162			+	+
**67**	dicaffeoyldimaloylquinic acid derivative	34.7	241, 306sh, 329	909	**747b**, 631, 585, 469, 353	631, 585, 469, 353	162			+	+
**68**	dicaffeoyldissuccinoylquinic acid	34.8	235, 306sh, 329	715	615, **553**, 515, 453b, 353	453b, 353, 191	162	+		+	
**69**	dicaffeoylmaloylquinic acid derivative	35.5	239sh, 307sh, 327	793	**631b**, 469, 353	469b, 451, 353, 191	162			+	+
**70**	tricaffeoylmaloylquinic acid	36.3	310sh, 326	793	**631b**, 469, 353, 277	469b, 353, 277, 191	162	+	+	+	+
**71**	dicaffeoylmaloylsuccinoylquinic acid derivative	38.2	238sh, 307sh, 328	893	**731b**, 631, 469, 353	631b, 469, 353	162			+	+
**72**	tricaffeoylsuccinoylquinic acid	38.5	241sh, 307sh, 325	777	**615**, 597, 515, 497b, 453, 353, 335	515, 453b, 353, 335	162			+	+
**73**	dicaffeoylquinic acid derivative	40.1	241sh, 305sh, 326	1071	**909**, 793b, 614, 515	793b, 613	162			+	+
**74**	dicaffeoyldimaloylquinic acid derivative	40.6	239sh, 305sh, 328	1071	**909b**, 748	747b, 632, 469, 353	162			+	+

b—base peak (the most abundant ion in the recorded spectrum); in bold—ions subjected to MS^2^ or MS^3^ fragmentation; NL—neutral loss detected corresponding to the cleavage of sugar or phenolic acid. * Identification based on a hierarchical key developed by Clifford [56,57]. ^#^ Comparisons with chemical standard have been made.

## Data Availability

The data presented in this study are available on request from the corresponding author.

## References

[1] Wink M., van Wyk B.E. (2004). Rośliny Lecznicze Świata.

[2] Sederski M.E. (2017). Prawie Wszystko o Ziołach i Ziołolecznictwie.

[3] Romagnolo D.F., Selmin O.I., Romagnolo D.F., Selmin O.I. (2016). Mediterranean Diet: Dietary Guidelines and Impact on Health and Disease.

[4] Lim T.K. (2015). Edible Medicinal and Non-Medicinal Plants.

[5] De la Rosa L.A., Alvarez-Parrilla E., Gonzalez-Aguilar G. (2010). Fruit and Vegetable Phytochemicals.

[6] Silva M., Silva L.R. (2015). Hydroxycinnamic Acids (HCAS): Structure, Biological Properties and Health Effects.

[7] Coman V., Vodnar D.C. (2019). Hydroxycinnamic acids and human health: Recent advances. J. Sci. Food Agric..

[8] Anioł-Kwiatkowska J., Kwiatkowski S., Berdowski W. (1993). Rośliny Lecznicze: Atlas.

[9] Ożarowski A., Jaroniewski W. (1987). Rośliny Lecznicze i Ich Praktyczne Zastosowanie.

[10] Carlotto J., Da Silva L.M., Dartora N., Maria-Ferreira D., Sabry D.D.A., Filho A.P.S., Werner M.F.d.P., Sassaki G.L., Gorin P.A.J., Iacomini M. (2015). Identification of a dicaffeoylquinic acid isomer from *Arctium lappa* with a potent anti-ulcer activity. Talanta.

[11] Carlotto J., de Souza L.M., Baggio C.H., Werner M.F.D.P., Maria-Ferreira D., Sassaki G.L., Iacomini M., Cipriani T.R. (2016). Polysaccharides from *Arctium lappa* L.: Chemical structure and biological activity. Int. J. Biol. Macromol..

[12] Liu W., Wang J., Zhang Z., Xu J., Xie Z., Slavin M., Gao X. (2014). In vitro and in vivo antioxidant activity of a fructan from the roots of *Arctium lappa* L.. Int. J. Biol. Macromol..

[13] Su S., Wink M. (2015). Natural lignans from *Arctium lappa* as antiaging agents in Caenorhabditis elegans. Phytochemistry.

[14] Chan Y.S., Cheng L.N., Wu J.H., Chan E., Kwan Y.W., Lee S.M.Y., Leung G.P.H., Yu P.H.F., Chan S.W. (2011). A review of the pharmacological effects of *Arctium lappa* (burdock). Inflammopharmacology.

[15] De Almeida A.B.A., Sánchez-Hidalgo M., Martín A.R., Luiz-Ferreira A., Trigo J.R., Vilegas W., Dos Santos L.C., Souza-Brito A.R.M., De La Lastra C.A. (2013). Anti-inflammatory intestinal activity of *Arctium lappa* L. (Asteraceae) in TNBS colitis model. J. Ethnopharmacol..

[16] Ferracane R., Graziani G., Gallo M., Fogliano V., Ritieni A. (2010). Metabolic profile of the bioactive compounds of burdock (*Arctium lappa*) seeds, roots and leaves. J. Pharm. Biomed. Anal..

[17] Haghi G., Hatami A., Mehran M. (2013). UPLC and HPLC of caffeoyl esters in wild and cultivated *Arctium lappa* L.. Food Chem..

[18] Jaiswal R., Kuhnert N. (2011). Identification and characterization of five new classes of chlorogenic acids in burdock (*Arctium lappa* L.) roots by liquid chromatography/tandem mass spectrometry. Food Funct..

[19] Kuo D.H., Hung M.C., Hung C.M., Liu L.M., Chen F.A., Shieh P.C., Ho C.T., Way T. (2012). Der Body weight management effect of burdock (*Arctium lappa* L.) root is associated with the activation of AMP-activated protein kinase in human HepG2 cells. Food Chem..

[20] Lin L.Z., Harnly J.M. (2008). Identification of hydroxycinnamoylquinic acids of arnica flowers and burdock roots using a standardized LC-DAD-ESI/MS profiling method. J. Agric. Food Chem..

[21] Liu S., Chen K., Schliemann W., Strack D. (2005). Isolation and identification of arctiin and arctigenin in leaves of burdock (*Arctium lappa* L.) by polyamide column chromatography in combination with HPLC-ESI/MS. Phytochem. Anal..

[22] Duh P. (1998). Antioxidant activity of burdock (*Arctium lappa* Linné): Its scavenging effect on free-radical and active oxygen. J. Am. Oil Chem. Soc..

[23] Wang B.S., Yen G.C., Chang L.W., Yen W.J., Duh P. (2007). Der Protective effects of burdock (*Arctium lappa* Linne) on oxidation of low-density lipoprotein and oxidative stress in RAW 264.7 macrophages. Food Chem..

[24] Ji K.Y., Jang J.H., Lee E.H., Kim S.M., Song H.W., Yang W.K., Kim H.Y., Kim K.H., Lee Y.S., Kim D.S. (2018). *Canavalia gladiata* and *Arctium lappa* extracts ameliorate dextran sulphate sodium-induced inflammatory bowel disease by enhancing immune responses. J. Funct. Foods.

[25] Wang Z., Li P., Wang C., Jiang Q., Zhang L., Cao Y., Zhong W., Wang C. (2016). Protective effects of *Arctium lappa* L. root extracts (AREs) on high fat diet induced quail atherosclerosis. BMC Complement. Altern. Med..

[26] Pomari E., Stefanon B., Colitti M. (2013). Effect of *Arctium lappa* (burdock) extract on canine dermal fibroblasts. Vet. Immunol. Immunopathol..

[27] Ghorat F., Azizkhani M., Naji S., Ranjbary A.G., Doostishoar F. (2017). Histopathological evaluation of Burdock (*Arctium lappa*) root hydroalcoholic extract on wound healing. Iran. Red Crescent Med. J..

[28] Lee C.J., Park S.K., Kang J.Y., Kim J.M., Yoo S.K., Han H.J., Kim D.O., Heo H.J. (2019). Melanogenesis regulatory activity of the ethyl acetate fraction from *Arctium lappa* L. leaf on α-MSH–induced B16/F10 melanoma cells. Ind. Crops Prod..

[29] Knott A., Reuschlein K., Mielke H., Wensorra U., Mummert C., Koop U., Kausch M., Kolbe L., Peters N., Stäb F. (2008). Natural *Arctium lappa* fruit extract improves the clinical signs of aging skin. J. Cosmet. Dermatol..

[30] Dos Santos A.C., Baggio C.H., Freitas C.S., Lepieszynski J., Mayer B., Twardowschy A., Missau F.C., dos Santos É.P., Pizzolatti M.G., Marques M.C.A. (2008). Gastroprotective activity of the chloroform extract of the roots from *Arctium lappa* L.. J. Pharm. Pharmacol..

[31] El-Kott A.F., Bin-Meferij M.M. (2015). Use of *Arctium lappa* Extract Against Acetaminophen-Induced Hepatotoxicity in Rats. Curr. Ther. Res. Clin. Exp..

[32] Lin S.C., Lin C.H., Lin C.C., Lin Y.H., Chen C.F., Chen I.C., Wang L.Y. (2002). Hepatoprotective effects of *Arctium lappa* Linne on liver injuries induced by chronic ethanol consumption and potentiated by carbon tetrachloride. J. Biomed. Sci..

[33] Da Silva L.M., Allemand A., Mendes D.A.G.B., dos Santos A.C., André E., de Souza L.M., Cipriani T.R., Dartora N., Marques M.C.A., Baggio C.H. (2013). Ethanolic extract of roots from *Arctium lappa* L. accelerates the healing of acetic acid-induced gastric ulcer in rats: Involvement of the antioxidant system. Food Chem. Toxicol..

[34] Da Silva L.M., Burci L.D.M., Crestani S., de Souza P., da Silva R.d.C.M.V.d.A.F., Dartora N., de Souza L.M., Cipriani T.R., da Silva-Santos J.E., André E. (2018). Acid-gastric antisecretory effect of the ethanolic extract from *Arctium lappa* L. root: Role of H+, K+-ATPase, Ca2+ influx and the cholinergic pathway. Inflammopharmacology.

[35] Liu H.C., Ku M.K., Chung F.Y., Lin C.C., Lin S.R. (2012). Effectiveness of great burdock essence compounds in the adjuvant treatment of gastric ulcer patients infected with *Helicobacter pylori*. Genomic Med. Biomark. Health Sci..

[36] Wu Y.C., Lin L.F., Yeh C.S., Lin Y.L., Chang H.J., Lin S.R., Chang M.Y., Hsiao C.P., Lee S.C. (2010). Burdock essence promotes gastrointestinal mucosal repair in ulcer patients. Fooyin J. Health Sci..

[37] Xu Z., Ju J., Wang K., Gu C., Feng Y. (2014). Evaluation of hypoglycemic activity of total lignans from *Fructus Arctii* in the spontaneously diabetic Goto-Kakizaki rats. J. Ethnopharmacol..

[38] Xu Z., Gu C., Wang K., Ju J., Wang H., Ruan K., Feng Y. (2015). Arctigenic acid, the key substance responsible for the hypoglycemic activity of *Fructus Arctii*. Phytomedicine.

[39] Lee Y.J., Choi D.H., Cho G.H., Kim J.S., Kang D.G., Lee H.S. (2012). *Arctium lappa* ameliorates endothelial dysfunction in rats fed with high fat/cholesterol diets. BMC Complement. Altern. Med..

[40] Wang Y., Zhang N., Kan J., Zhang X., Wu X., Sun R., Tang S., Liu J., Qian C., Jin C. (2019). Structural characterization of water-soluble polysaccharide from *Arctium lappa* and its effects on colitis mice. Carbohydr. Polym..

[41] Zhang X., Zhang N., Kan J., Sun R., Tang S., Wang Z., Chen M., Liu J., Jin C. (2020). Anti-inflammatory activity of alkali-soluble polysaccharides from *Arctium lappa* L. and its effect on gut microbiota of mice with inflammation. Int. J. Biol. Macromol..

[42] Lou Z., Wang H., Lv W., Ma C., Wang Z., Chen S. (2010). Assessment of antibacterial activity of fractions from burdock leaf against food-related bacteria. Food Control.

[43] Lou Z., Wang H., Tang Y., Chen X. (2017). The effect of burdock leaf fraction on adhesion, biofilm formation, quorum sensing and virulence factors of *Pseudomonas aeruginosa*. J. Appl. Microbiol..

[44] Tian X., Sui S., Huang J., Bai J.P., Ren T.S., Zhao Q.C. (2014). Neuroprotective effects of *Arctium lappa* L. roots against glutamate-induced oxidative stress by inhibiting phosphorylation of p38, JNK and ERK 1/2 MAPKs in PC12 cells. Environ. Toxicol. Pharmacol..

[45] Tian X., Guo L.P., Hu X.L., Huang J., Fan Y.H., Ren T.S., Zhao Q.C. (2015). Protective Effects of *Arctium lappa* L. Roots Against Hydrogen Peroxide-Induced Cell Injury and Potential Mechanisms in SH-SY5Y Cells. Cell. Mol. Neurobiol..

[46] Kwon Y.K., Choi S.J., Kim C.R., Kim J.K., Kim Y.J., Choi J.H., Song S.W., Kim C.J., Park G.G., Park C.S. (2016). Antioxidant and cognitive-enhancing activities of *Arctium lappa* L. roots in Aβ1-42-induced mouse model. Appl. Biol. Chem..

[47] Rutkowski L. (1998). Klucz do Oznaczania roślin Naczyniowych Polski Niżowej.

[48] European Medicines Agency (2010). Community Herbal Monograph on Arctium lappa L., Radix.

[49] Šarić-Kundalić B., Dobeš C., Klatte-Asselmeyer V., Saukel J. (2010). Ethnobotanical study on medicinal use of wild and cultivated plants in middle, south and west Bosnia and Herzegovina. J. Ethnopharmacol..

[50] Jarić S., Kostić O., Mataruga Z., Pavlović D., Pavlović M., Mitrović M., Pavlović P. (2018). Traditional wound-healing plants used in the Balkan region (Southeast Europe). J. Ethnopharmacol..

[51] Zhou X., Zhang H., Ge L., Gong H., Tian S. (2011). Determination of arctiin and arctigenin contents in *Arctium tomentosum* Mill. by HPLC method. J. Chem..

[52] Strawa J., Jakimiuk K., Waluk M., Poslednik M., Nazaruk J., Tomczyk M. (2020). Phytochemical examination of wolly burdock *Arctium tomentosum* leaves and flower heads. Chem. Nat. Compd..

[53] Halliwell B. (1987). Disease: Some New Concepts. FASEB J..

[54] Sugiura Y., Torii T., Matsuda K., Yamada Y. (2009). Anti-allergic effects of extracts from commercial products of cooked burdock. Food Sci. Technol. Res..

[55] Chagas-Paula D.A., Oliveira T.B., Faleiro D.P.V., Oliveira R.B., Da Costa F.B. (2015). Outstanding Anti-inflammatory Potential of Selected Asteraceae Species through the Potent Dual Inhibition of Cyclooxygenase-1 and 5-Lipoxygenase. Planta Med..

[56] Clifford M.N., Johnston K.L., Knight S., Kuhnert N. (2003). Hierarchical scheme for LC-MSn identification of chlorogenic acids. J. Agric. Food Chem..

[57] Clifford M.N., Knight S., Kuhnert N. (2005). Discriminating between the six isomers of dicaffeoylquinic acid by LC-MSn. J. Agric. Food Chem..

[58] Bazylko A., Piwowarski J.P., Filipek A., Bonarewicz J., Tomczyk M. (2013). In vitro antioxidant and anti-inflammatory activities of extracts from *Potentilla recta* and its main ellagitannin, agrimoniin. J. Ethnopharmacol..

[59] Choi C.W., Kim S.C., Hwang S.S., Choi B.K., Ahn H.J., Lee M.Y., Park S.H., Kim S.K. (2002). Antioxidant activity and free radical scavenging capacity between Korean medicinal plants and flavonoids by assay-guided comparison. Plant Sci..

[60] Schepetkin I.A., Kirpotina L.N., Jakiw L., Khlebnikov A.I., Blaskovich C.L., Jutila M.A., Quinn M.T. (2009). Immunomodulatory Activity of Oenothein B Isolated from *Epilobium angustifolium*. J. Immunol..

[61] O’Dowd Y., Driss F., Dang P.M.C., Elbim C., Gougerot-Pocidalo M.A., Pasquier C., El-Benna J. (2004). Antioxidant effect of hydroxytyrosol, a polyphenol from olive oil: Scavenging of hydrogen peroxide but not superoxide anion produced by human neutrophils. Biochem. Pharmacol..

[62] Wang D., Bădărau A.S., Swamy M.K., Shaw S., Maggi F., Da Silva L.E., López V., Yeung A.W.K., Mocan A., Atanasov A.G. (2019). *Arctium* Species Secondary Metabolites Chemodiversity and Bioactivities. Front. Plant Sci..

